# Identification of human annexin A6 as a novel cellular interactant of influenza A M2 protein: implications for influenza life cycle

**Published:** 2011-01-10

**Authors:** HL Ma, F Kien, Y Zhang, J Tse, LLM Poon, B Nal

**Affiliations:** 1 HKU-Pasteur Research Centre, Hong Kong, Hong Kong SAR; 2 Department of Microbiology, Li Ka Shing Faculty of Medicine, The University of Hong Kong, Hong Kong SAR; 3 Department of Anatomy, Li Ka Shing Faculty of Medicine, The University of Hong Kong, Hong Kong SAR

## Background

During its replication, influenza virus utilizes the host cellular machineries for many aspects of its life cycle. Characterization of such virus-host protein-protein interactions is a must to identify determinants of pathogenesis. The M2 ion channel protein plays a crucial role during the entry and late stages of the viral life cycle where its C-terminal domain, well conserved among influenza A viruses, is accessible to cellular machineries after fusion with endosomal membrane and during its trafficking along the secretory pathway prior to assembly and budding. The aim of the study is to identify cellular interactants of M2 that play important regulatory roles during influenza infection.

## Methods

To identify cellular partners of M2 we performed a genome-wide yeast-two-hybrid (Y2H) screening approach using the cytosolic domain of M2 as bait and a human placenta random primed cDNA library as prey and tested more than 60 million interactions.

## Results

From the Y2H screening, an interesting interaction with the human annexin A6 (ANXA6) protein, a member of annexin family proteins that binds to phospholipds in a Ca^2+^-dependent manner was identified. Co-immunoprecipitation of myc-tagged ANXA6 and viral M2 proteins co-expressed in HEK293T cells after transfection and infection confirmed the direct interaction between ANXA6 and M2. We further investigated whether this interaction had any functional significance with regards to influenza life cycle. Using a RNA interference strategy to silence the ANXA6 gene in human lung epithelial A549 cells, we observed increased progeny virus titers either in a single or multiple viral growth kinetics study suggesting a negative regulatory role for ANAX6 during viral infection.

## Conclusion

A novel interaction between M2 and ANXA6 was identified. More functional studies are in progress to define precisely the potential negative regulatory role of this interaction during viral infection. A systematic dissection of the viral life cycle will be performed to identify the step(s) affected by the ANXA6 cellular factor using specific assays such as real-time quantitative RT-PCR in a single or multiple viral growth kinetics study, cell transduction with HA- and M2-pseudotyped lentiviral particles, virion attachment and internalization assay, immunofluorescence staining of NP protein as a marker of viral ribonucleoproteins localization, viral polymerase activity measurement and viral budding observation by electron microscopy.

